# Supplementary material for: Molecular dissection of colorectal cancer in pre-clinical models identifies biomarkers predicting sensitivity to EGFR inhibitors
Source: Nat Commun. 2017 Feb 10;8:14262. doi: 10.1038/ncomms14262 (PMC5309787; doi:10.1038/ncomms14262)
Supplement: Supplementary Information — Supplementary Figures and Supplementary Tables [file ncomms14262-s1.pdf]

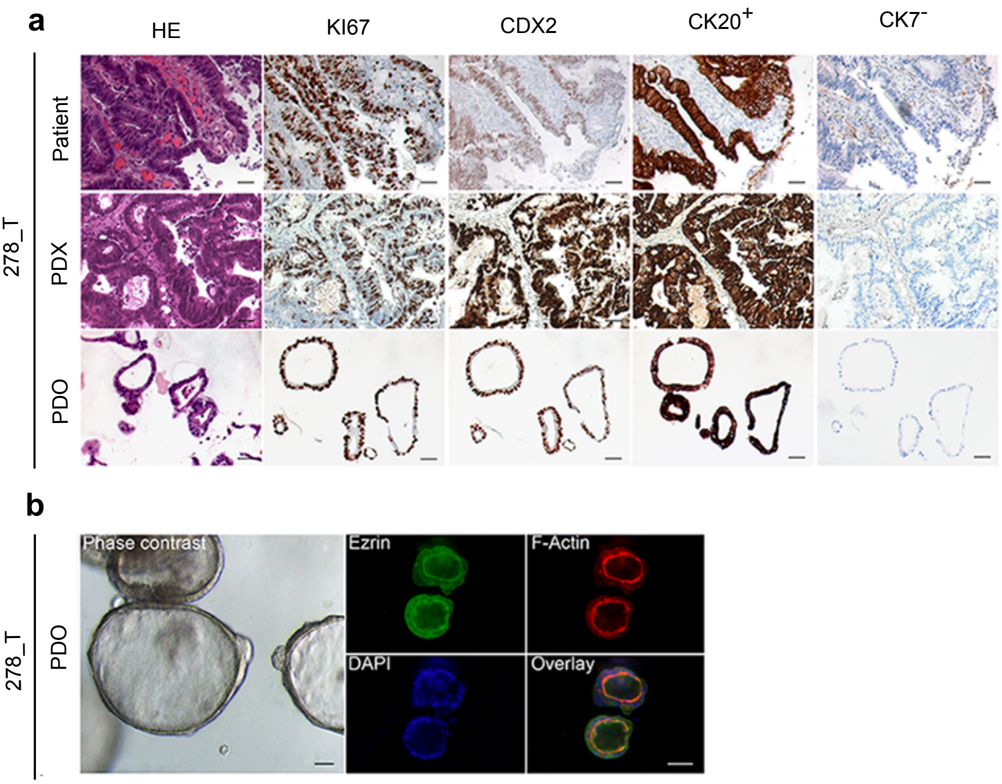

Supplementary Figure 1: Representative example of phenotypic and morphological characterization of CRC primary tissue, PDX and PDO models for 278_T. **a)** Comparison of immunohistological staining between a differentiated primary tumour and the derived PDX and PDO model (278_T), stained for hematoxylin-eosin, CDX2 and $CK20^{+}/CK7^{-}$. KI67 expression marks actively proliferating cells in both tissue and culture derivative. **b)** Immunohistological staining of 278_T_CELL is characterized by generating sphere-like structures that stain for Ezrin (green), F-actin (red) and nuclei (blue). Scale bars for all images: 50 µm.

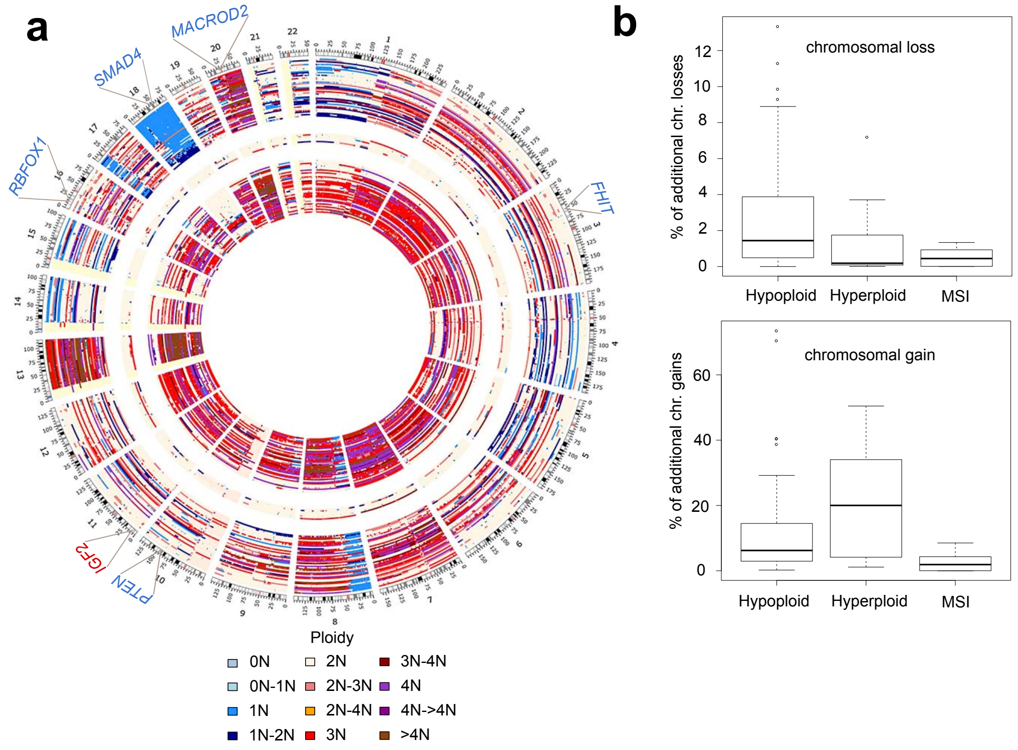

Supplementary Figure 2: Copy number variations in the OT cohort.
a) Circos plot representing the chromosome ideograms of tumours and metastases (tumour purity of ≥35%). Three sample groups with characteristic CNV patterns are shown: hypoploid (outer circle, *n*=43), chromosomally stable MSI (middle circle, *n*=8) and hyperploid (inner circle, *n*=40). Recurrently amplified (*IGF2*, red) or deleted genes (*SMAD4*, *MACROD2*, *FHIT*, *PTEN* and *RBFOX1,* blue) are noted. **b)** Distribution of additional chromosomal losses and gains in models compared to the original tumour for each CNV group (hypoploid: *n*=28, hyperploid: *n*=21, MSI: *n*=6). Note the significant loss of genetic material in models derived from hypoploid tumours (p-value = 0.005, t-test) and the gain in models derived from hyperploid samples (P = 0.04, t-test).

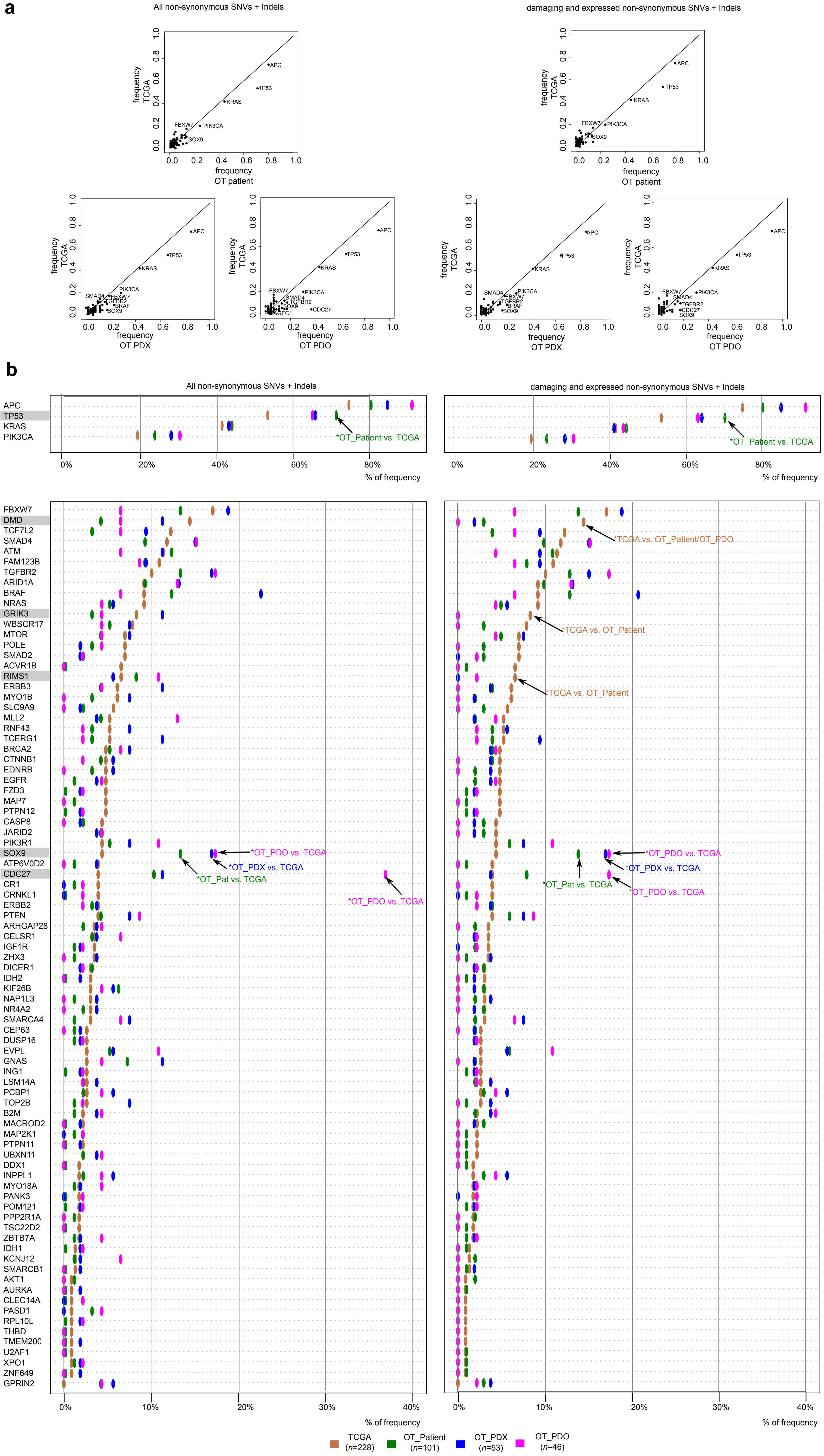

Supplementary Figure 3: Frequency of non-synonymous SNVs and Indels in the OT patient-, PDX-and PDO-cohort compared to TCGA 2012. On the left all detected non-synonymous SNVs and on the right damaging and expressed non-synonymous SNVs were considered for the OT cohorts (tumour purity ≥40%; see methods). Cancer-relevant genes are shown, mentioned in the TCGA or PAN study, or recurrently mutated genes in OT (see methods). **a)** Scatter plots showing mutation frequencies in OT and in TCGA. Genes with a frequency >15% in either data set are labeled. **b)** Dotplot of mutation frequencies in TCGA (orange), OT patient (green), OT_PDX (blue) and OT_PDO (pink) are shown. Significant differences in frequencies between TCGA and OT are marked by arrows and grey a background (binomial test; FDR < 0.05).

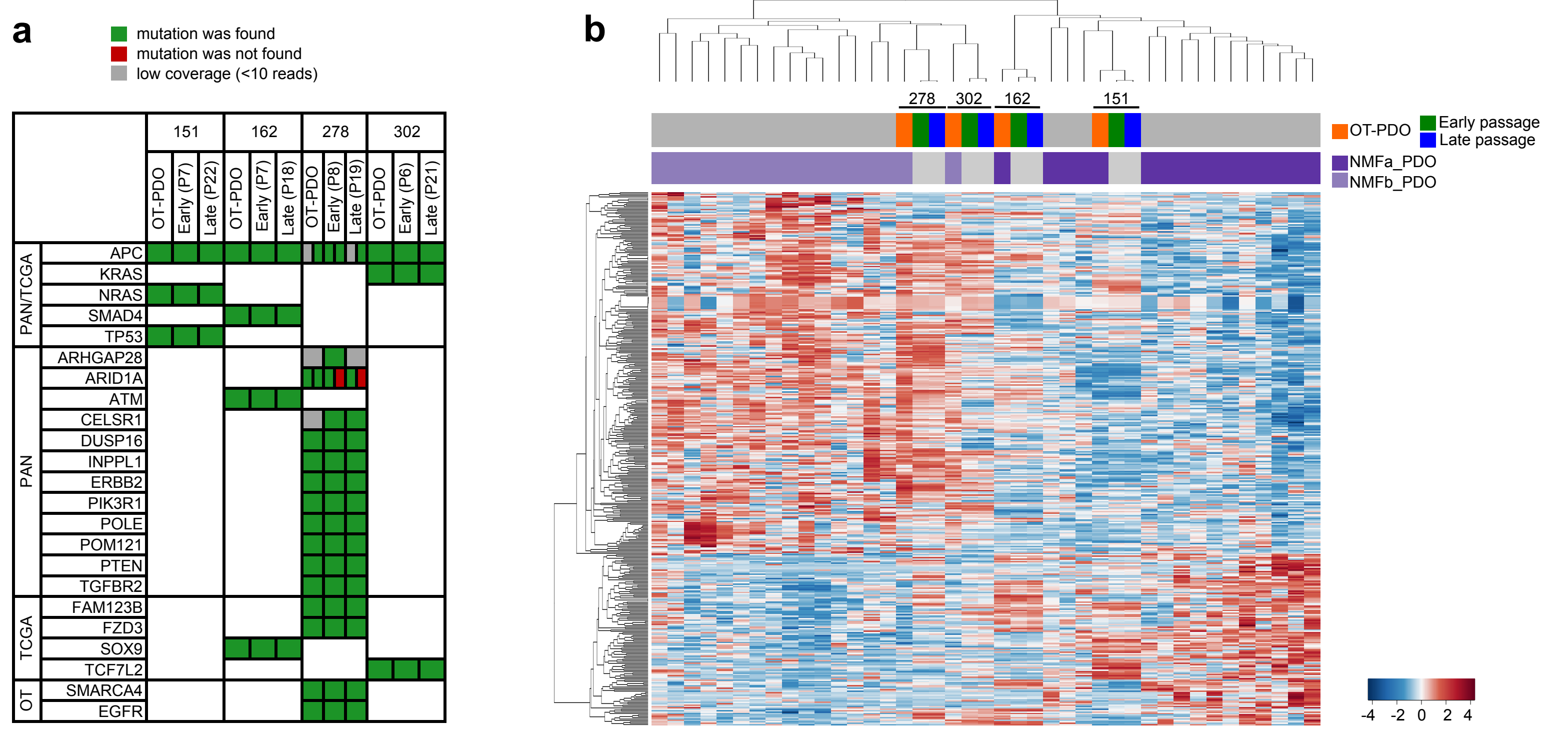

Supplementary Figure 4: Stability of gene expression profiles and somatic alterations between early and late passage PDO models.
a) Comparison of CRC relevant somatic alterations between early and late passage models and the matching OT-PDO sample is shown as indicated in the caption. Numbers in brackets indicate the passage number. Only cancer-relevant genes are shown, selected from TCGA, PAN, or OT recurrently mutated genes resources (see methods). **b)** Hierarchical clustering of 33 OT-PDO models and four early and late passage models (278,302,162,151) based on the genes defining NMFa_PDO and NMFb_PDO molecular groups. Early passage, late passage and matching OT-PDO models are marked as indicated in the caption. Numbers of passages are shown in panel b.

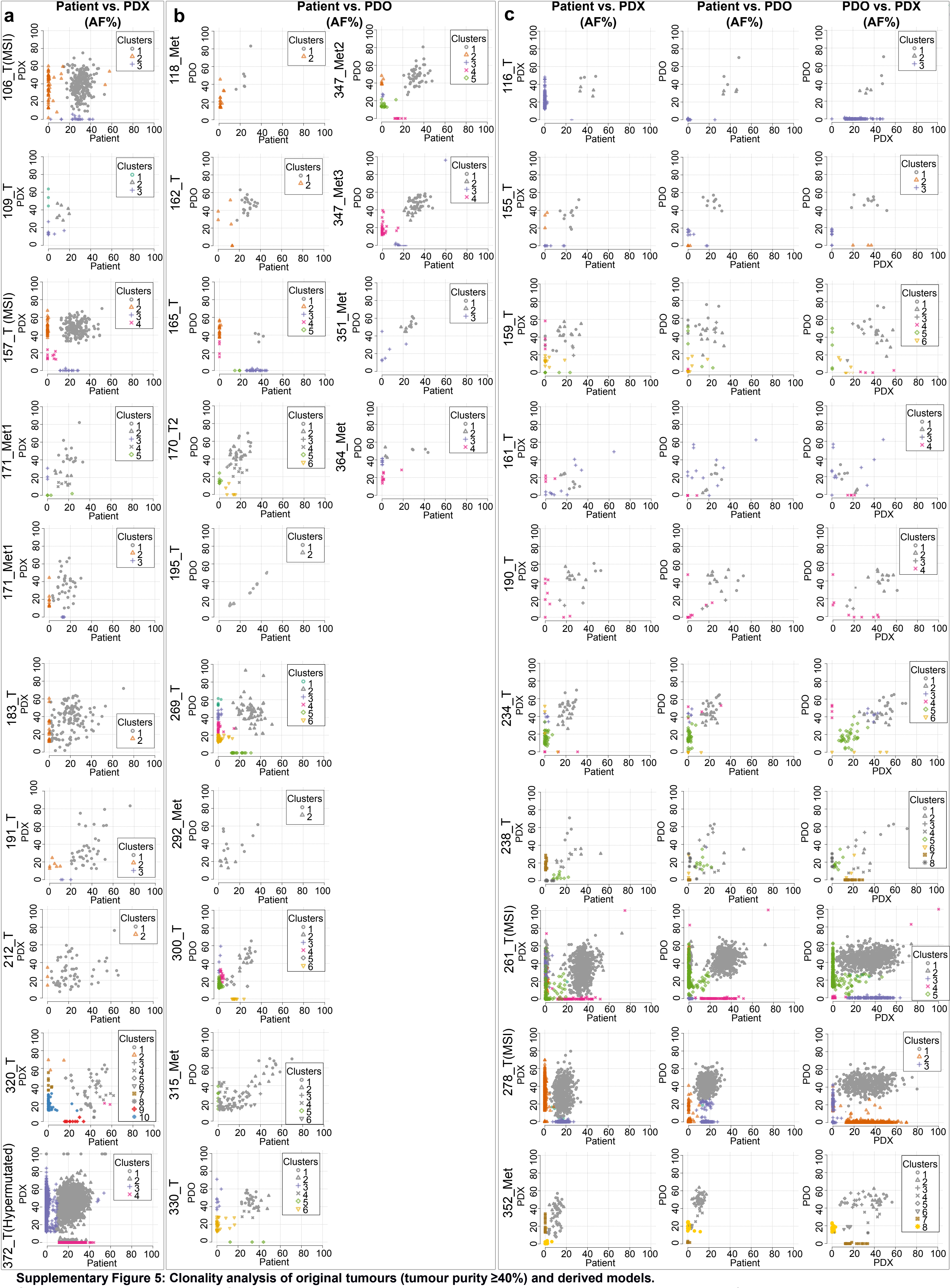

Supplementary Figure 5: Clonality analysis of original tumours (tumour purity ≥40%) and derived models.
Clonality analysis is based on the sciClone algorithm. The plots display the mutations in diploid regions clustered by their allele frequencies (AF). Individual mutation clusters were shown by different colours where grey indicated the mutations found in common between the compared samples. Samples that passed the sciClone analysis are shown. MSI and hypermutated samples are marked. **a)** Patient tumours with only a PDX (*n*=10); **b)** patient tumours with only a PDO (*n*=14) and **c)** patient tumours with both PDX and PDO (*n*=12, without 327_T and 323_T those two are shown in Figure 3a).

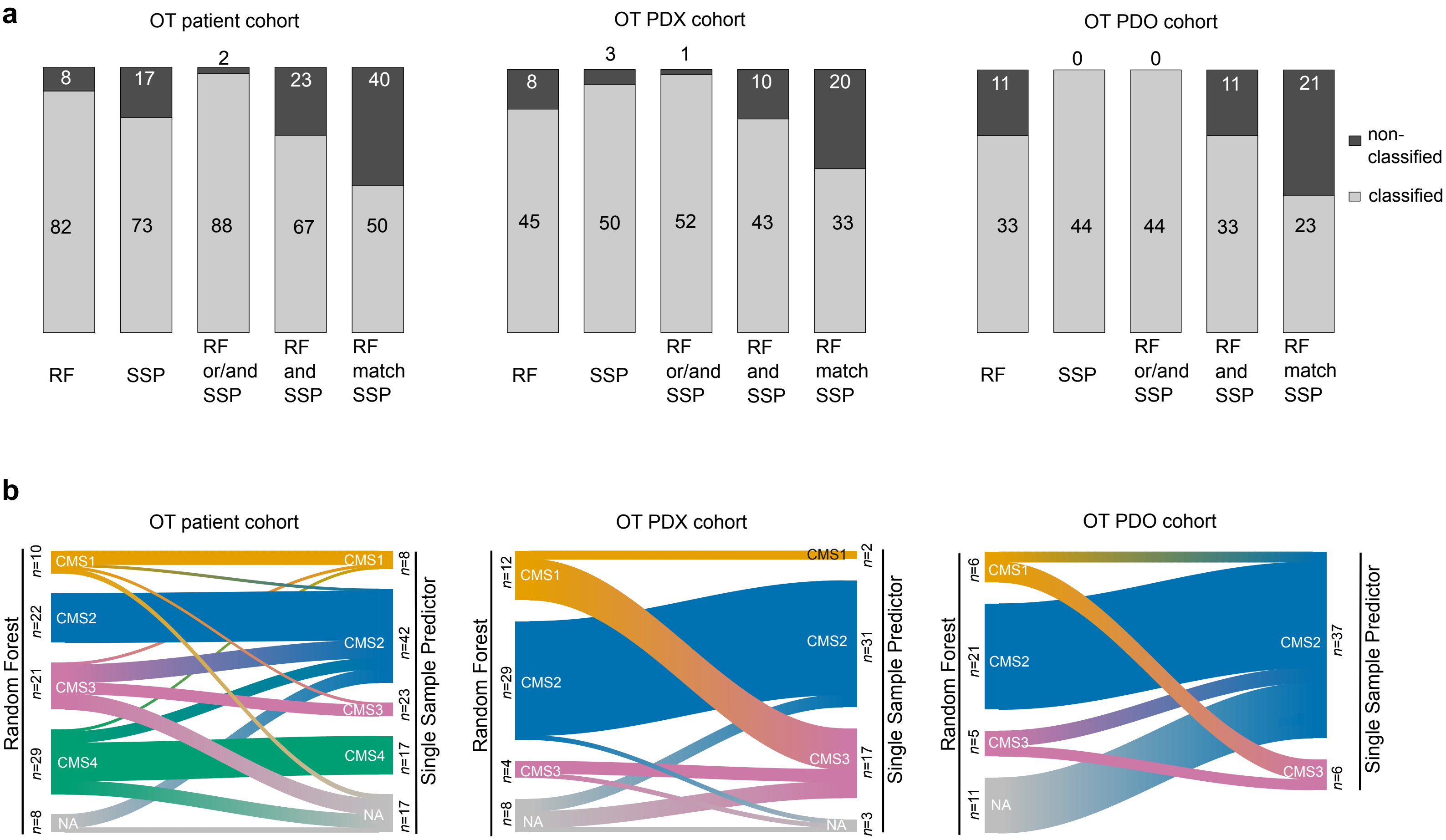

Supplementary Figure 6: RF and SSP CMS classification on the OT cohort (tumour purity ≥40%).
a) Bar plots show the results and the comparison of CMS classifiers random forest (RF) and single sample prediction (SSP) applied on the OT patient tumours (left), PDX (middle) and PDO cohort (right). Light grey areas show the fraction of samples that fulfil the following criteria: "RF" – samples classified by RF, "SSP" – samples classified by SSP, "RF or/and SSP" – samples classified by at least one of the two classifiers, "RF and SSP" – samples classified by both classifiers, "RF match SSP" – samples concordantly classified by both classifiers. Dark grey areas show the fraction of samples that do not fulfil these criteria. Sample numbers are indicated on the plot. **b)** Sankey plots show the correspondence of the RF and SSP classification on the OT patient tumour (left), PDX (middle) and PDO cohort (right).

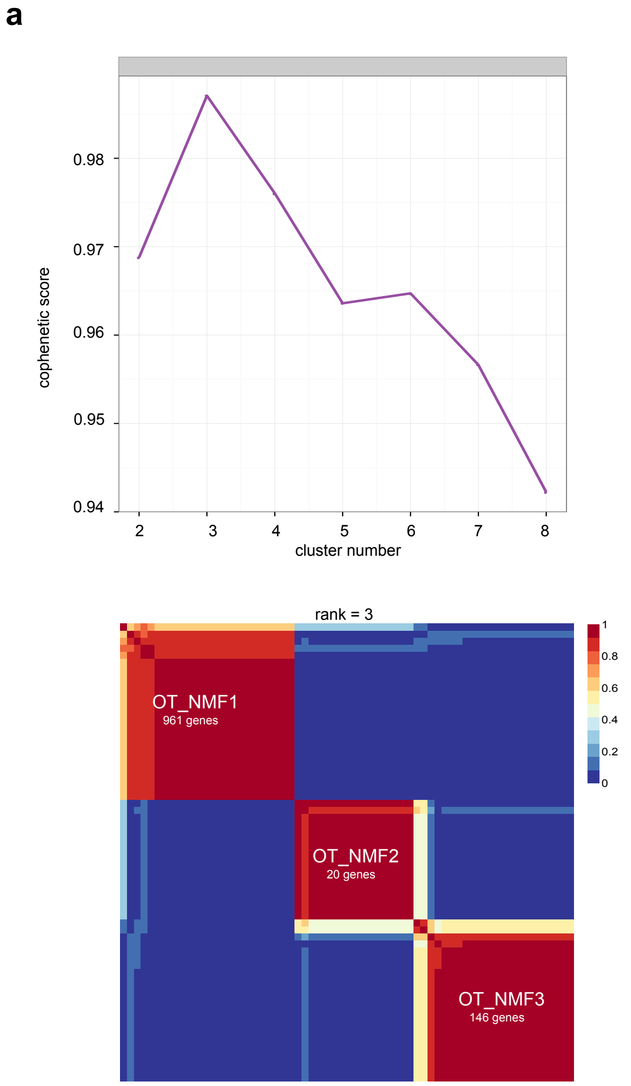

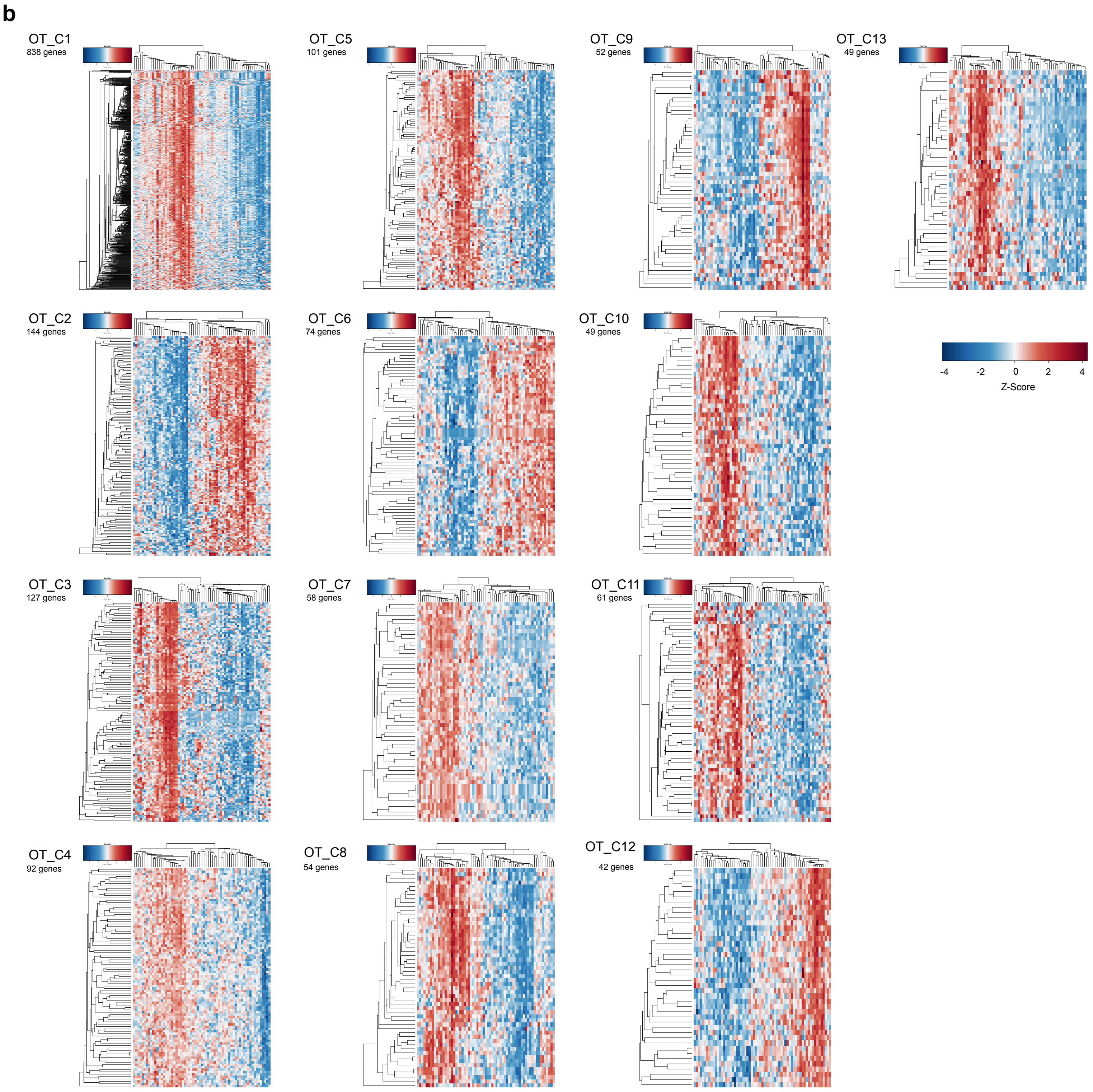

Supplementary Figure 7: Clustering results of NMF and CLICK algorithms applied on the OT patient cohort (tumour purity ≥40%).
a) Results of the NMF clustering. Upper graph shows the cophenetic scores for the tested cluster sizes 2-8. The lower panel shows the consensus matrix for the cluster of three groups. Number of genes determining each cluster is noted. **b)** Results of the CLICK analysis. Hierarchical clustering of co-expressed genes in each of the 13 CLICK clusters in the OT cohort is shown (see methods). The number of genes in each cluster is indicated.

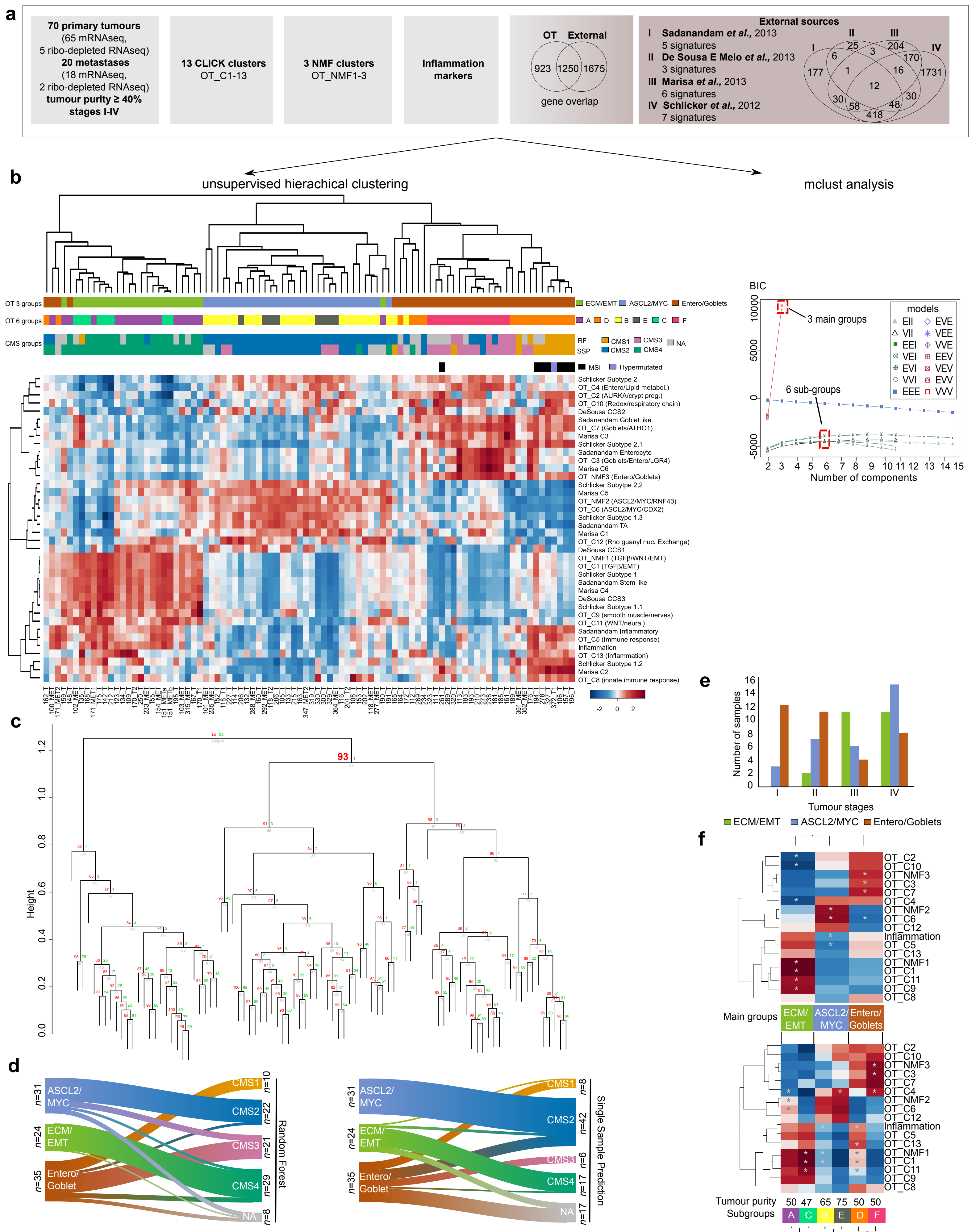

Supplementary Figure 8: Analysis of the molecular groups identified in the OT patient cohort (tumour purity ≥40%).
a) Overview of 16 signatures corresponding to the OT clusters, a set of inflammation genes and 21 external signatures. Venn diagrams show the gene overlaps between OT and external signatures or between the four external sources. **b)** Unsupervised hierarchical clustering of the mean patterns of 38 CRC-related signatures in 90 patients (left). Information on molecular groups is indicated as follows from top to bottom under the dendogram. Colour labels for the three OT patient tumour groups, the six subgroups (A-F) and CMS groups are indicated in the caption on the right side. MSI- and hypermutated samples are marked with black and blue boxes, respectively. Mclust identifies either three or six subgroups of tumours in the OT cohort based on Bayesian Information Criterion (BIC) (right, see methods). **c)** The stability of the hierarchical clustering of the mean pattern matrix was tested with the R package pvclust (see methods). The order of the samples corresponds to Supplementary Fig. 5b). **d)** Sankey plots showing the correspondence between the three OT main groups and the four CMS classes (left: RF; right: SSP). **e)** Distribution of the tumour stages in the three main groups. **f)** Differentially expressed OT gene signatures. Heat maps displaying the mean of single sample gene set enrichment scores (ssGSES) for each NMF and CLICK signature in the three main groups (upper panel: ECM/EMT, ASCL2/MYC, Entero/Goblets) or in the six subgroups (lower panel: A-F). Asterisks indicate statistically significant differences (FDR ≤ 0.01) in the ssGSESs between the main groups (top) or in pair-wise comparison between two subgroups (A vs. C, B vs. E and D vs. F; bottom). The average tumour purity in each subgroup is indicated. Biological features of the molecular subgroups are summarized in Supplementary Table 10.

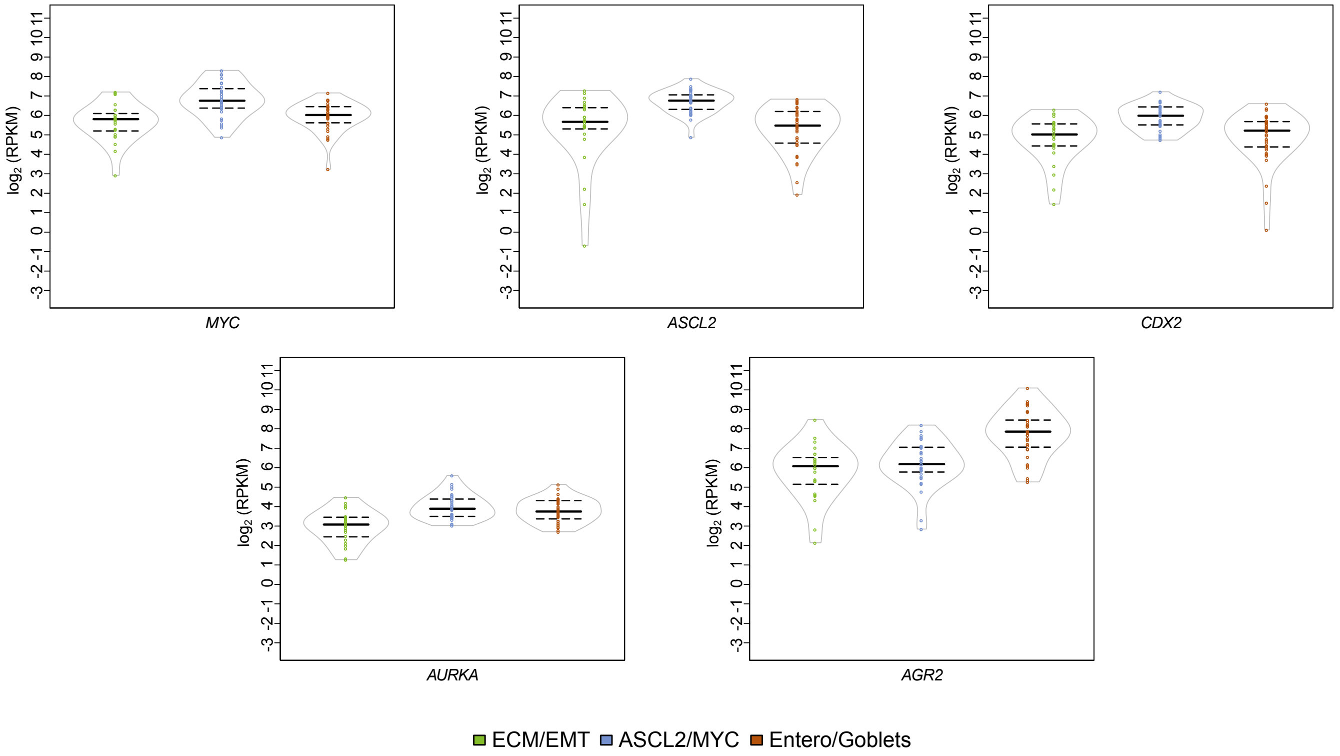

Supplementary Figure 9: Violin plots showing the expression of selected genes across the OT cohort (tumour purity ≥40%). Expression (log_2(RPKM)) of *MYC, ASCL2, CDX2, AURKA* and *AGR2* in the three main groups ECM/EMT, ASCL2/MYC and Entero/Goblets. Lines mark the 25%, 50% and 75% quantiles.

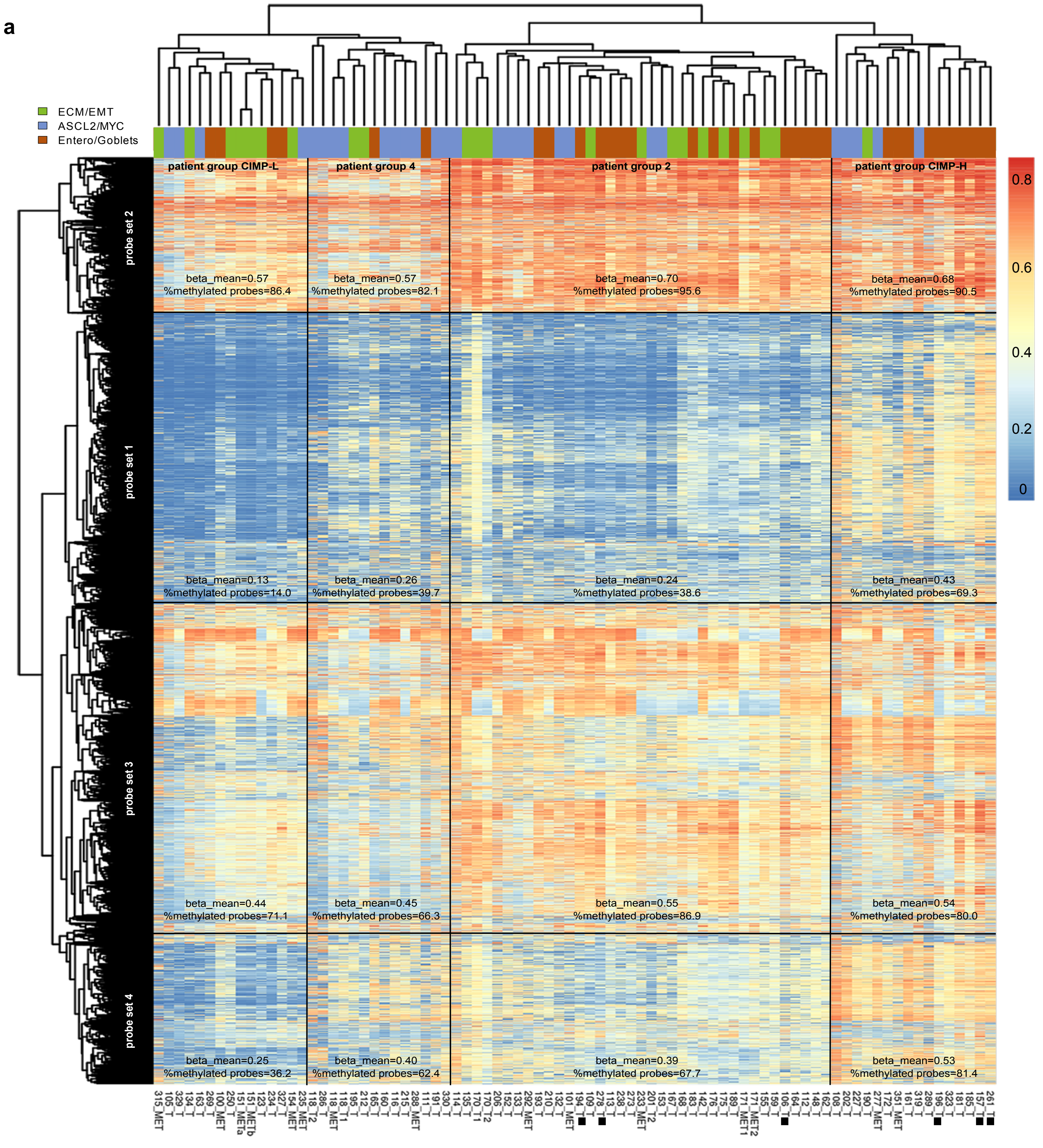

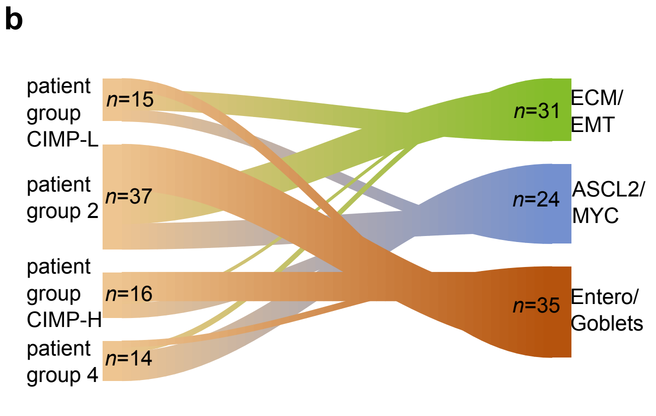

Supplementary Figure 10: DNA methylation clusters in the OT patient cohort (tumour purity ≥40%).

a) Hierarchical clustering of the patient tumours according to DNA methylation data. The primary tumour cohort was used to identify the top 5% most variable probes by median absolute deviation (MAD) (*n*= 22,358). These probes were used to cluster both metastatic and primary samples. Methylation per probe is expressed as beta value and given as a continuous colour code with no/low methylation in blue to high methylation in red. Four different probe sets were identified: probe set 1 (*n*= 7,025) localized mostly in gene promoters and in CpG islands (CGI's) and defined the CpG Island Methylator Phenotype (CIMP) identifying the CIMP-Low and CIMP-High tumour groups, displaying also pervasive CIMP; probe set 2 (*n*= 3,759) and probe set 3 (*n*= 7,858) corresponded to low CpG density loci either in intergenic regions and gene bodies or within 3'UTRs, respectively; probe set 4 (*n*= 3,716) was less defined, albeit slightly enriched for promoters in CGIs and CGI shores. The two additional patient groups 2 and 4 were defined by DNA methylation patterns involving probe sets 1, 2 and 3. The most methylated tumour group was CIMP-H, followed by patient group 2, then by group 4 and CIMP-L exhibiting the least methylation. Four major groups of tumours could be identified: CIMP-L (*n*=15), CIMP-H (*n*=16), patient group 2 (*n*=37) and patient group 4 (*n*=14). The three main molecular groups (ECM/EMT, ASCL2/MYC and Entero/Goblets) are coloured above the heatmap. MSI samples are marked at the bottom and cluster in the CIMP-H and patient 4 group. **b)** Sankey plot showing the correspondence from the four methylation groups into the three OT main groups (ECM/EMT, ASCL2/MYC and Entero/Goblets).

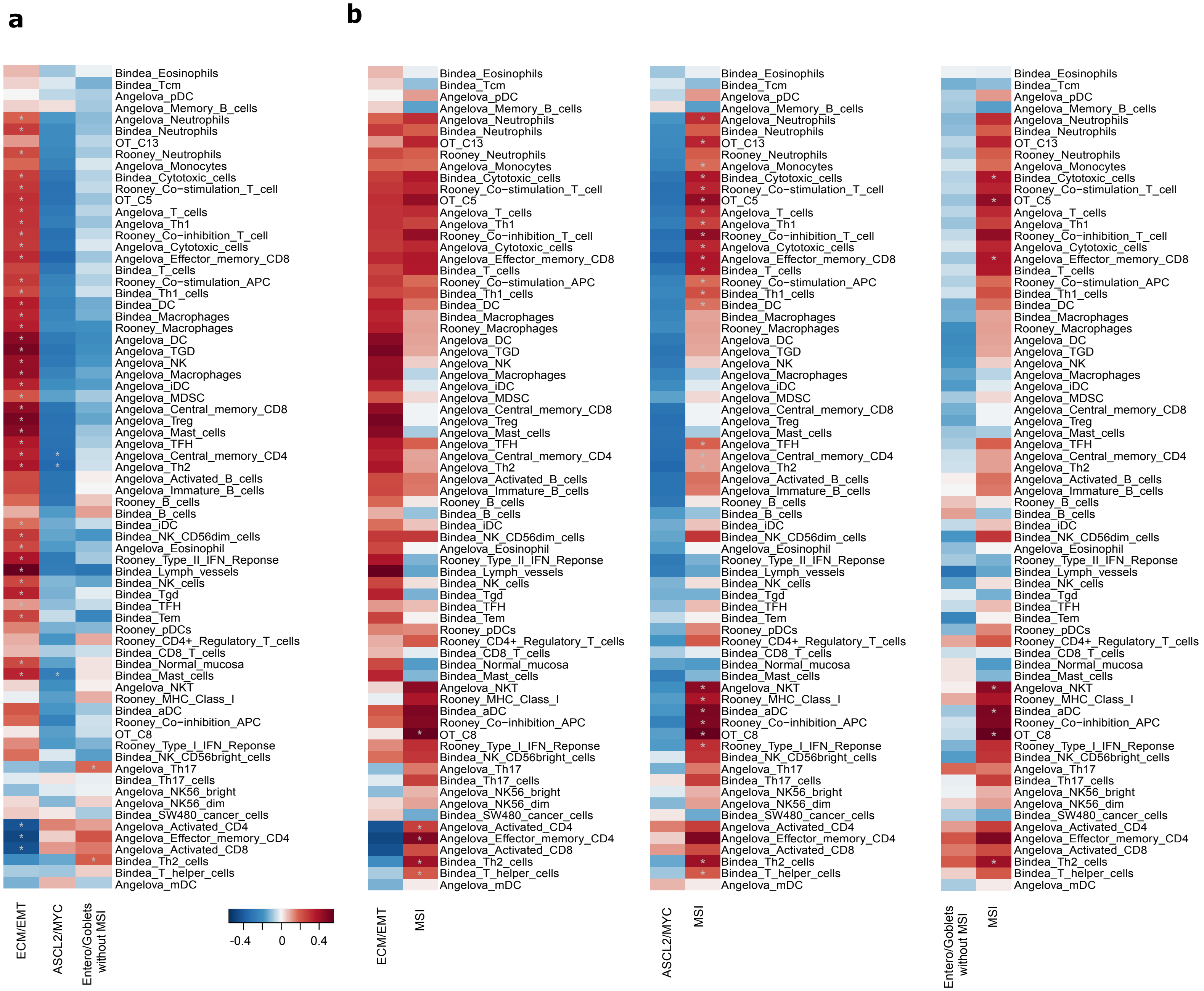

Supplementary Figure 11: Profiling of immune signatures in the OT cohort (tumour purity ≥40%). Mean gene set enrichment scores (GSES) for signatures of immune cell types/processes in the main molecular OT-groups. Asterisks indicate statistically significant differences in the GSESs (FDR ≤ 0.05). **a)** Comparison of ECM/EMT, ASCL2/MYC and Entero/Goblets (excluding MSI) samples. **b)** Pair-wise comparison between the MSI samples and each of the main molecular groups.

a

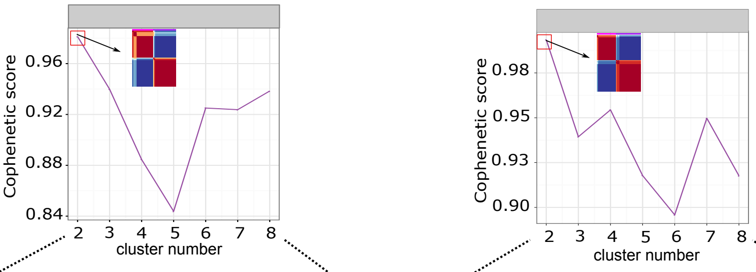

b

	PDO-NMFa	PDO-NMFb	PDX-NMFa	PDX-NMFb
**PROCESSES**	**WNT signaling** (p-value= 10^{-7})	**cellular glucuronidation response to xenobiotic stimulus** (p-value= 10^{-11})	**WNT signaling** & (p-value= 10^{-4})	**matrix metalloproteinase** (p-value = 10^{-5})
	**TGFβ** (p-value=10^{-4})	**cellular response to interferon** (p-value= 10^{-10})	**triglyceride homeostasis** (p-value= 10^{-6})	**carbohydrate metabolism** (p-value= 10^{-5})
	**stem cell processes** (p-value=10^{-9})	**lipid metabolism** (p-value= 10^{-5})	**β-catenin degradation** (p-value= 10^{-3})	
**TISSUES**	**mesoderm** (p-value=10^{-9})	**colon epithelial cells** (p-value= 10^{-14})	**colon** (p-value= 10^{-6})	**colon epithelial cells** (p-value = 10^{-16})
	**embryonic structures** (p-value=10^{-6})	**colon goblet cells** (p-value=10^{-9})		**colon goblet cells** (p-value=10^{-14})
**selected highly expressed GENES**	*PROX1; TGFβ2; CTNNB1; SHH; PTCH1; BAMBI; FGF3; FGF20; SMAD9; WNT5B; DACH1; PDGFC*	*UGT1A1; UGT1A3; UGT1A4; MX1; IFIT1; IFIT2; IFIT3; IRF7; IRF9; MUC1; MUC2; RCG4;CREB3L1*	*WIF1; TCF7; LGR5; PROX1; ASCL2; TGFβ1*	*AGR2; KLF4; TFF1; TFT2; SPDEF; MUC1; MUC2; MUC4; CREB3L1; KRT*

Supplementary Figure 12: Transcriptome analysis of the PDO and PDX models. **a)** Results of the NMF clustering. The graph shows the cophenetic scores for the tested clusters 2-8. Within the graph the consensus matrix for the cluster size two is shown for PDO (left) and PDX models (right). **b)** Functional annotation of NMF groups in PDO and PDX models using the GePS Genomatix software.

a

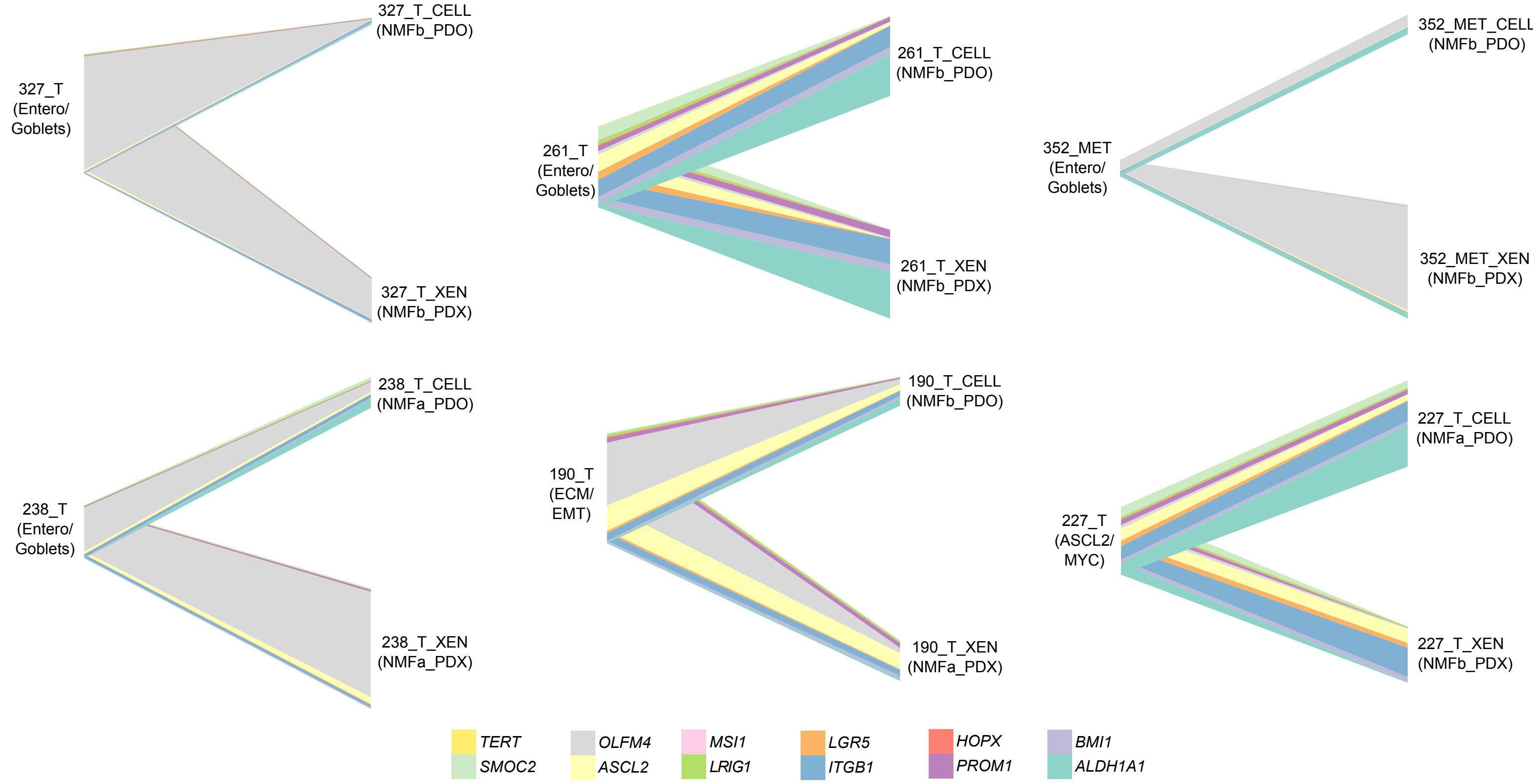

b

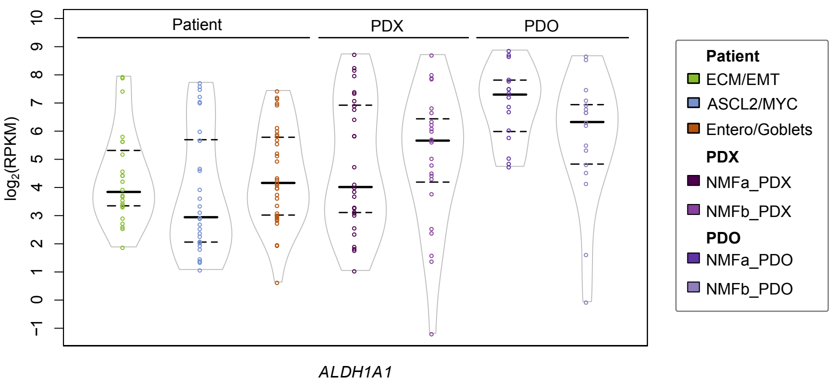

Supplementary Figure 13: Expression of known stem cell markers in the OT cohort (tumour purity ≥40%).
a) River plots displaying the expression differences in patient tumours compared to the models (PDX and PDO) in six trios (327_T, 261_T, 352_MET, 238_T, 190_T and 227_T). The corresponding OT main group (patient tumours) and NMFa/b group (models) are indicated. **b)** Violin plot showing the expression of *ALDH1A1* in patient tumours, PDX and PDO.

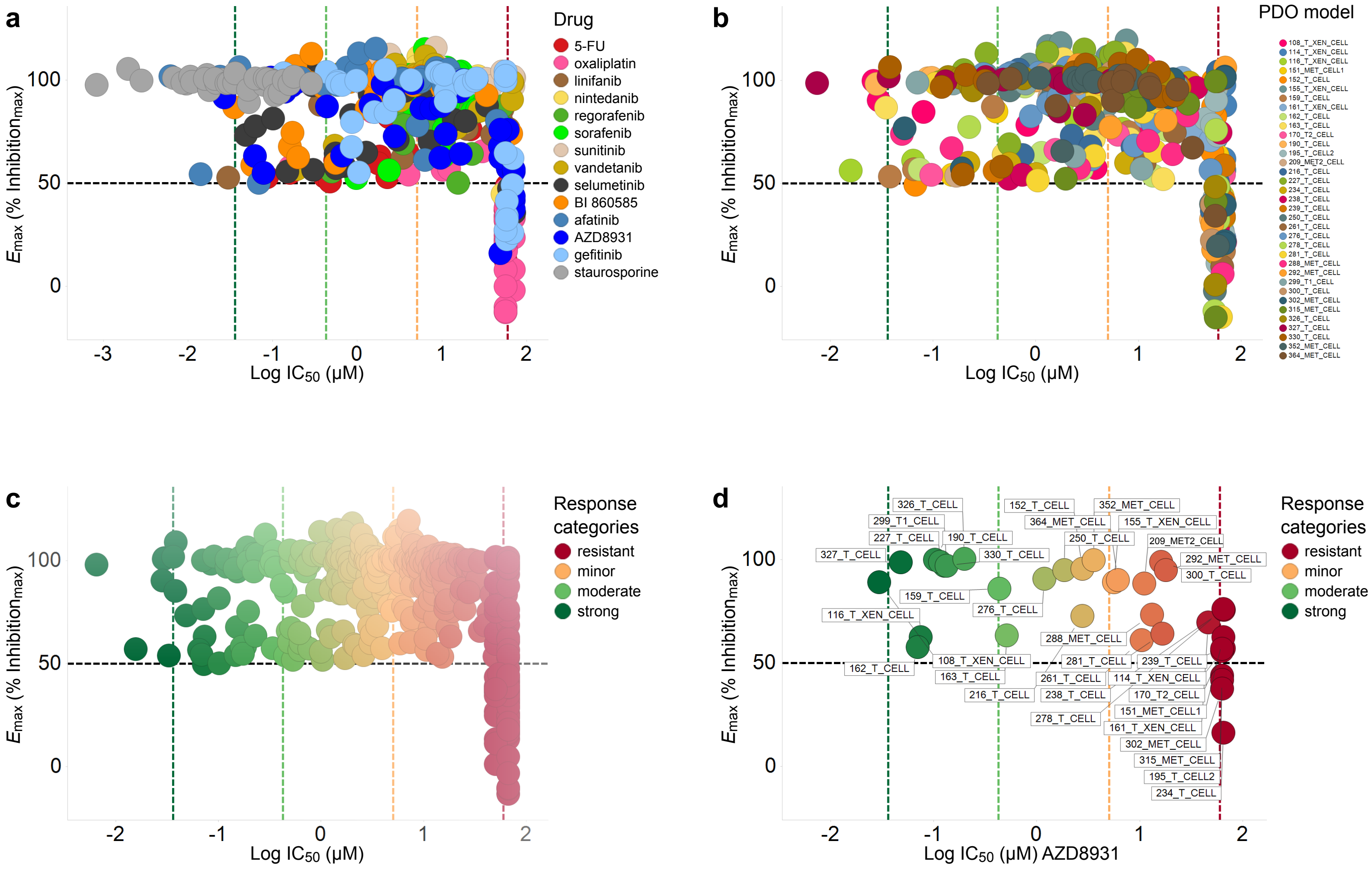

Supplementary Figure 14: Comparison of dose-response parameters in PDO models.
(a,b,c,d) Scatter plot of E_{max} (y-axis) and IC_{50} values (x-axis) across the PDO panel for all drugs including the reference compound staurosporine (a), for all drugs excluding the reference compound (b-c) and for AZD8931 (d). The horizontal dashed line represents 50% of E_{max}, and the vertical lines represent the thresholds for the response categories. Colours represent different drugs (a), different PDO models (b) or different responder groups (c-d).

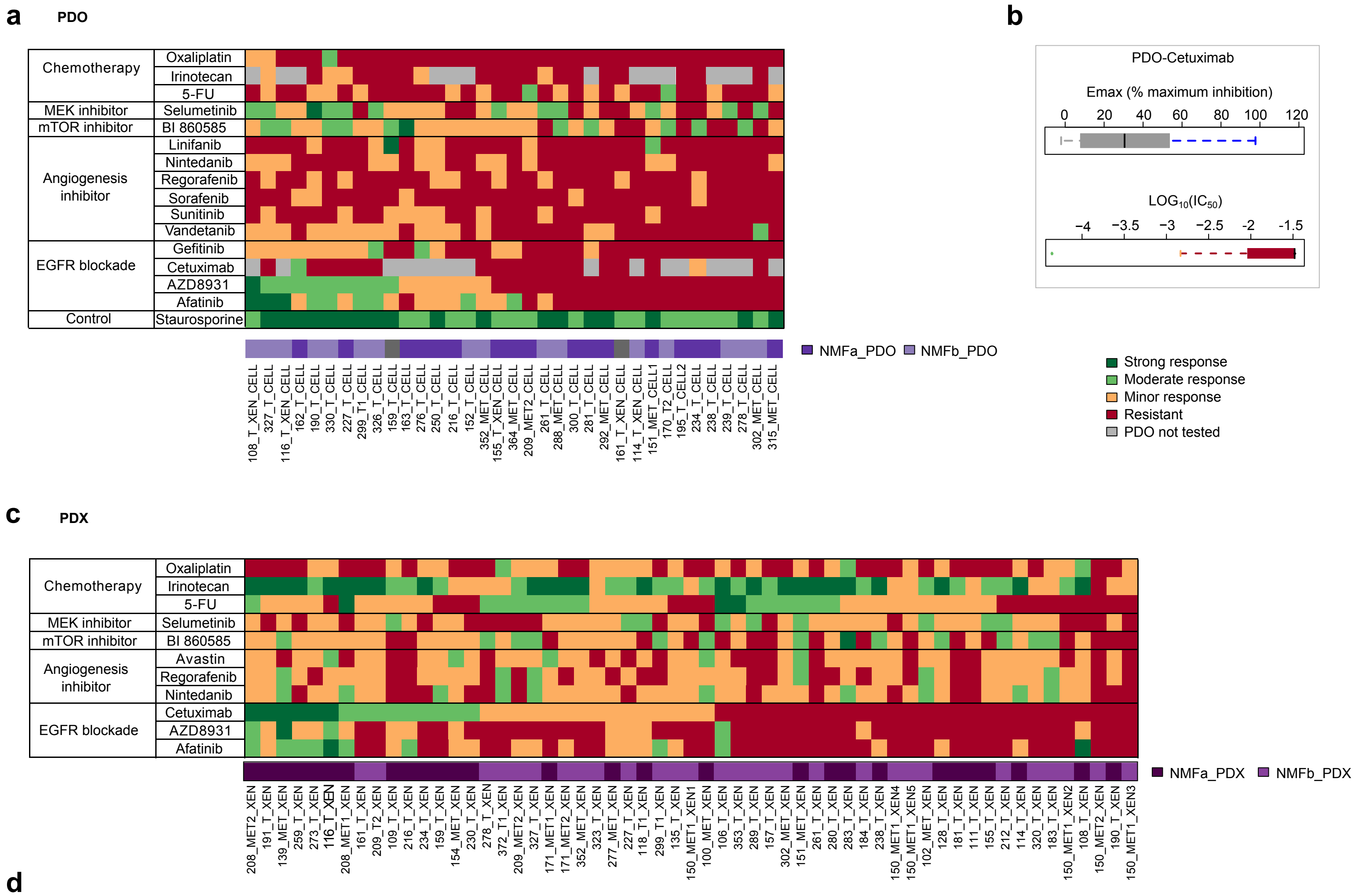

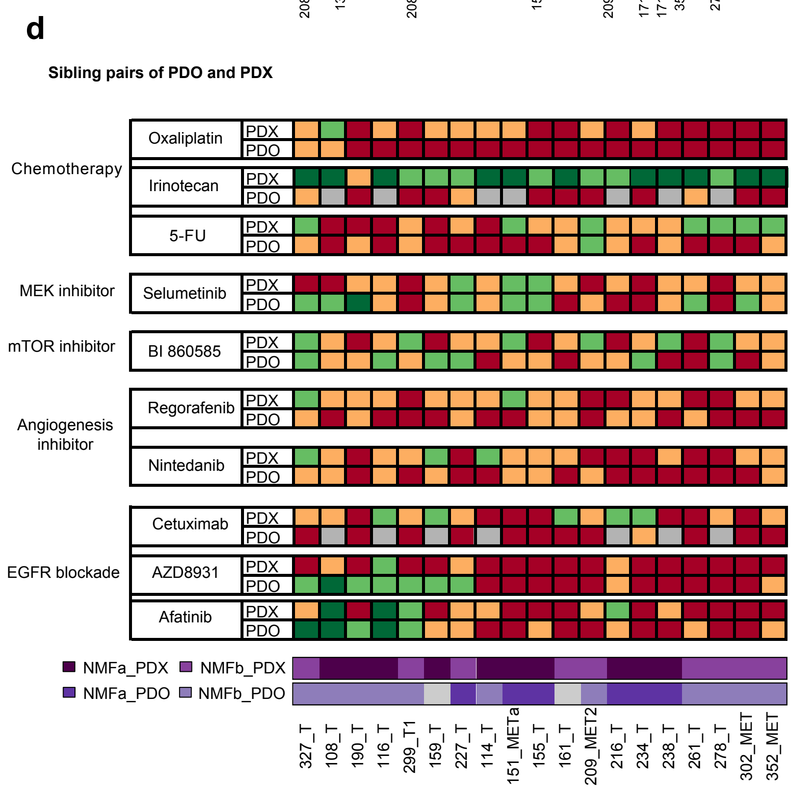

Supplementary Figure 15: Overview of drug response data in PDX and PDO models. Four response categories were defined for both PDX and PDO models: resistant, minor-, moderate- and strong response based on T/C (PDX) and IC_{50} (PDO) (see methods). The corresponding NMFa/b groups of PDX and PDO are indicated. In PDOs, irinotecan and cetuximab were tested on 21 and 19 models, respectively. Missing response values are marked in grey. **a)** Drug response of 35 PDO models to 15 drugs and staurosporine as a control. **b)** Maximum inhibition and potency of cetuximab in 19 PDOs. Upper box plot showing the maximum inhibition (E_{max}). Lower box plot showing the potency ($log_{10}(IC_{50})$). Range of drug efficiency (E_{max} > 50%) and inactivity (E_{max} < 50%) is indicated in blue and grey, respectively. The four response categories are coloured as indicated. **c)** Drug response of 59 PDXs to 11 drugs. **d)** Drug response of 19 sibling pairs of PDX and PDO models derived from the same tumours to ten drugs used in both model systems.

a

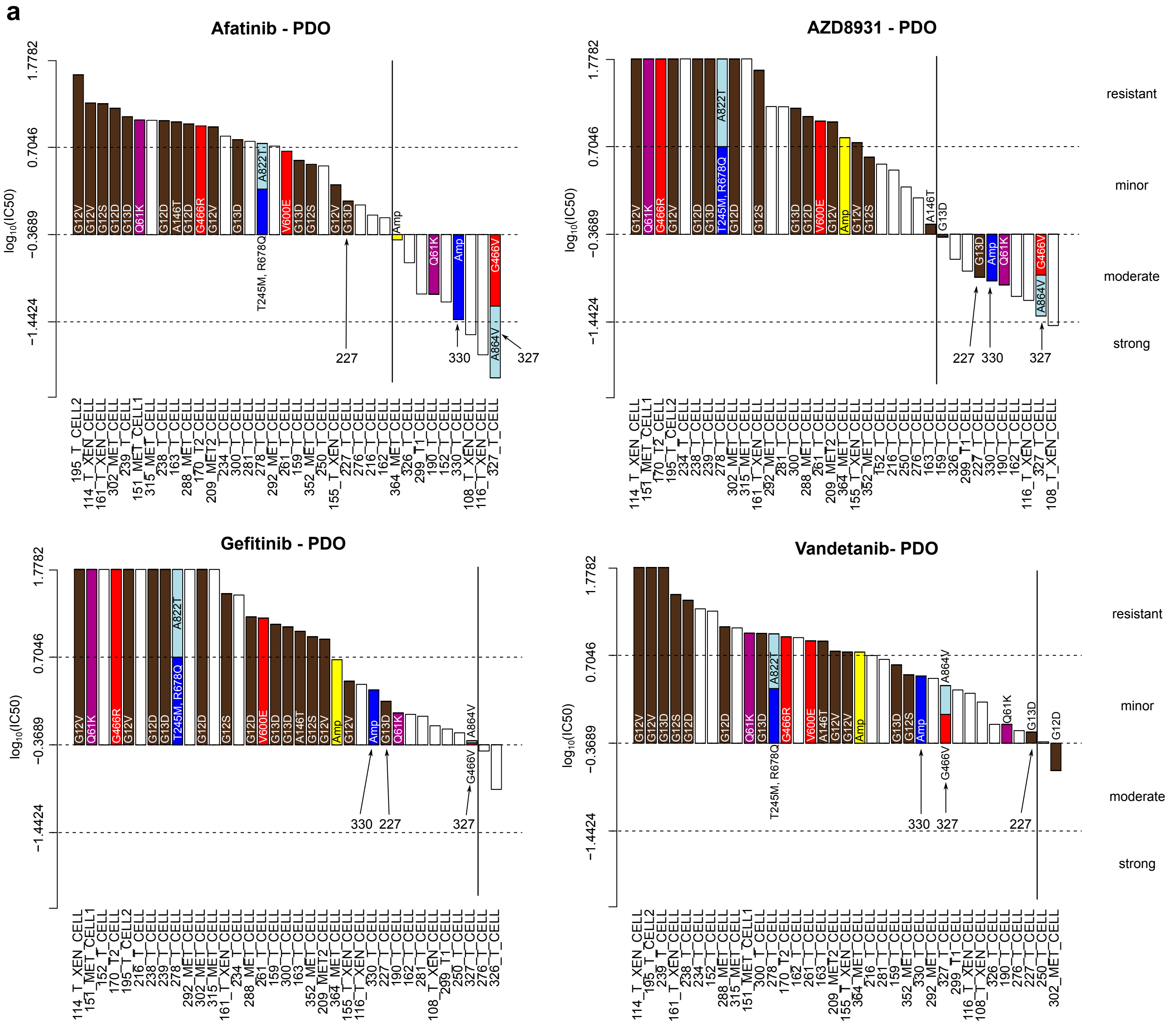

b

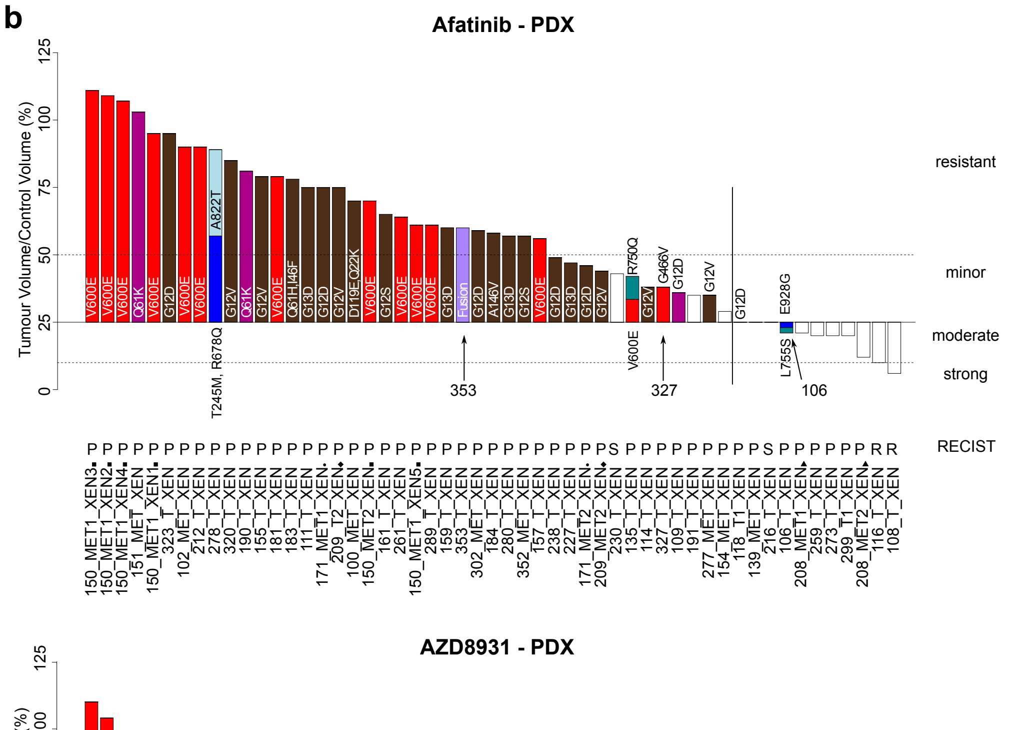

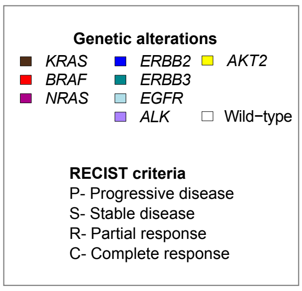

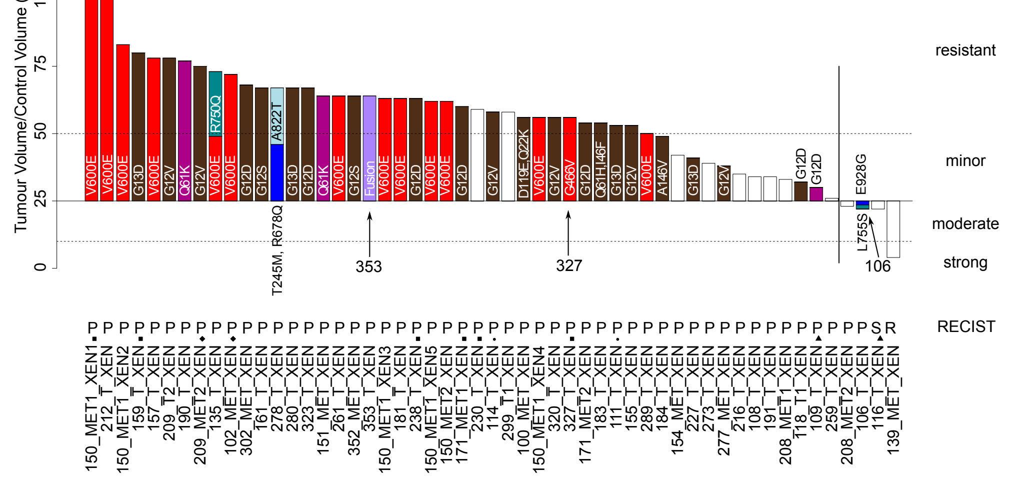

Supplementary Figure 16: Waterfall plots showing the drug sensitivity for EGFR-targeting drugs. Mutations and fusions in RAS/RAF and RTK pathways are shown. The vertical line shows the cut-off between responders and non-responders. **a)** Drug sensitivity of 35 PDO for AZD8931, afatinib, gefitinib and vandetanib is shown by the $\log_{10}(IC_{50})$. **b)** Drug sensitivity of 53 PDX for afatinib and AZD8931 are shown by T/C. The values corresponding to the RECIST criteria are indicated; models derived from the same patient are labelled with the same symbol above each sample name.

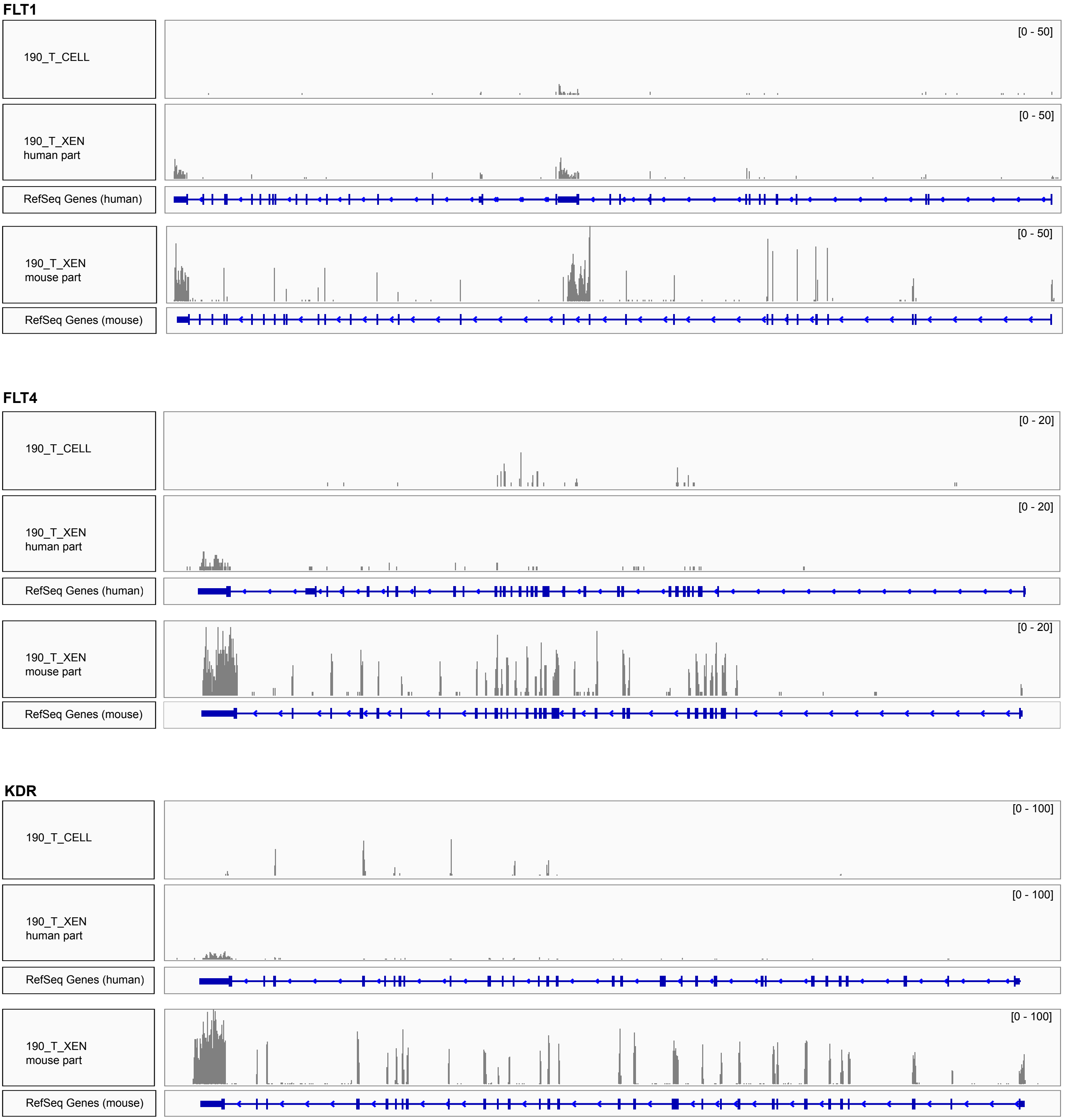

Supplementary Figure 17: Representative example of RNAseq read coverage for VEGF receptors. The expression of the three VEGF receptors (*FLT1*, *FLT4* and *KDR*) in the PDO models, PDX-human component and PDX-mouse component of tumour 190_T are shown as read counts on the IGV browser along with the RefSeq reference gene models for man and mouse.

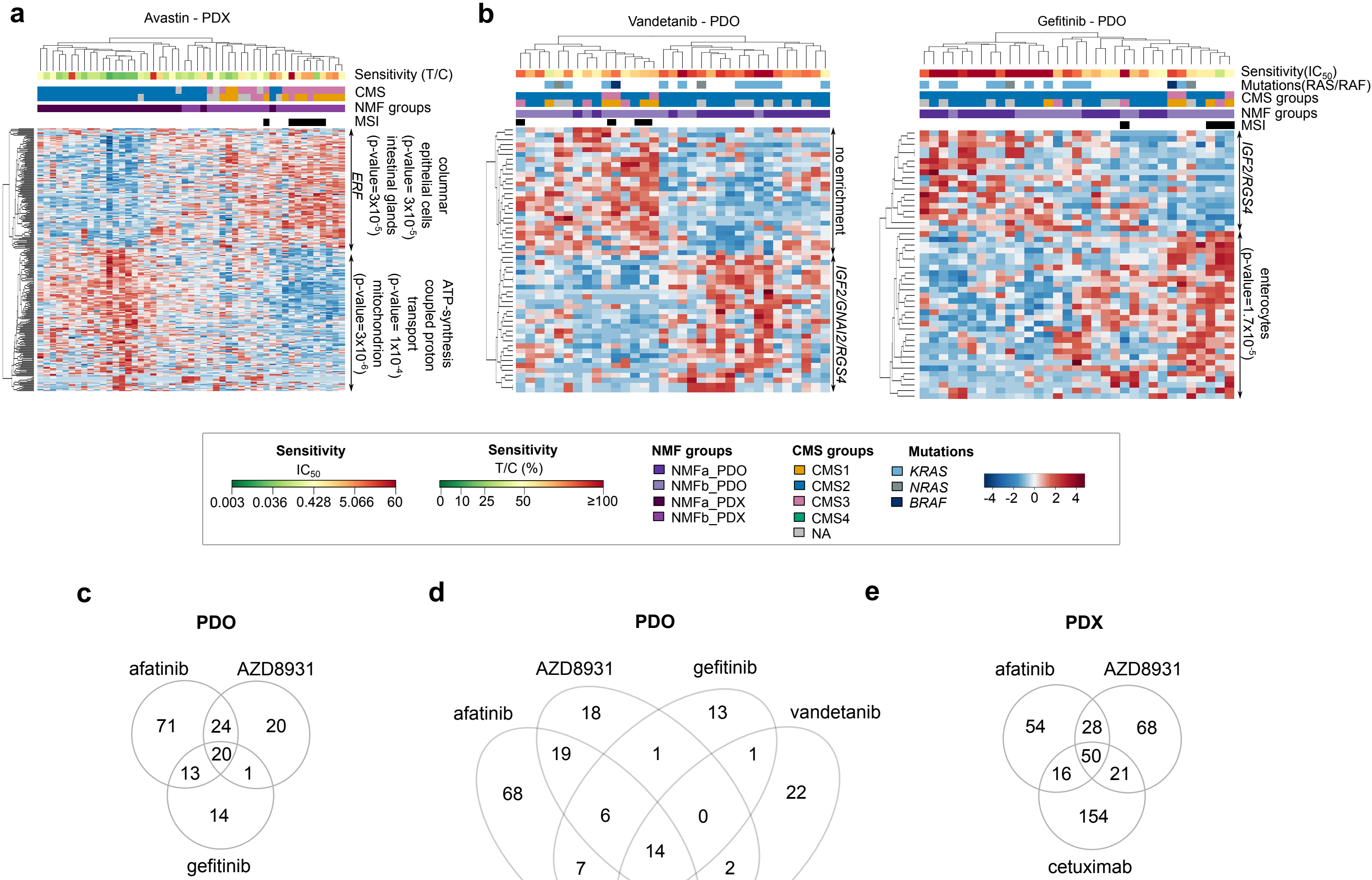

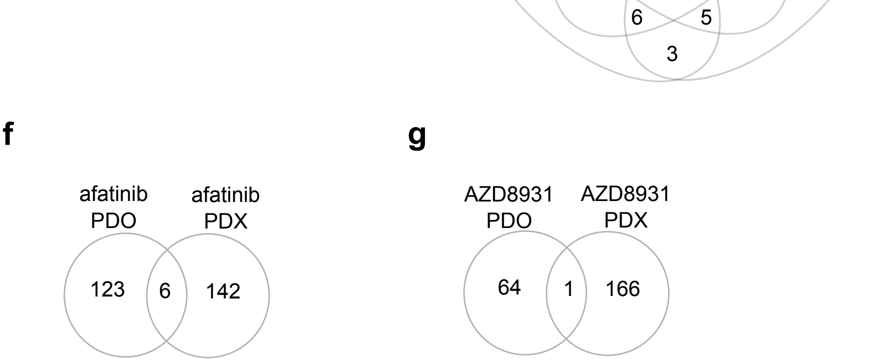

Supplementary Figure 18 : Comparison of gene signatures associated with response to Avastin, vandetanib and gefitinib in PDOs or PDXs.
a and b) Gene signature associated with drug sensitivity, represented by T/C (Avastin - PDX) or IC_{50} (vandetanib and gefitinib - PDO) continuous values coloured as indicated in the caption (dark green: strong response, red: resistance). MSI samples, OT-NMF-groups and CMS classifications (RF: upper row, SPP: bottom row) and mutation status are coloured as indicated in the caption. Avastin is shown in a) and vandetanib and gefitinib in b). **c,d,e)** Gene overlaps between signatures of the EGFR inhibitors. In c) and d) the signatures are based on drug sensitivity data from PDO models and in e) from PDX models. **f and g**) Genes in the drug signatures of afatinib (f) and AZD8931 (g) are compared between PDX and PDO models.

Supplementary Table 1: Respective performances of the OT mini-classifier and of the RAS/RAF mutation status in predicting cetuximab sensitivity. Following cohorts OT-PDX (OT) (cross-validation), EPO PDX, the Gao et al., 2015 PDX (NV) and Khambata-Ford et al., 2007 primary tumours (KF) were used. The mutation status was defined by mutations in codon 12 and 13 of KRAS or detected activating mutations in KRAS, BRAF or NRAS (BRAF mutations: V600E; KRAS/NRAS mutations: G12, G13, Q22, Q61, A146). In an additional setup, stable disease (SD) samples were excluded. Sensitivity, specificity and balanced accuracy were calculated based on true positive (TP), true negative (TN), false positive (FP) and false negative (FN) annotations and values ≥0.8 are marked in bold. d) Same as in (c) but taking as validation cohort 164 samples merged from OT, EPO, NV and KF.

Cohorts	Classifier	Samples analyzed	TP	FP	FN	TN	Sensitivity	Specificity	Balanced accuracy
OT (n=48)	Mutation status in *KRAS/BRAF/NRAS*	whole cohort (n=48)	10	6	4	28	0.71	**0.82**	0.77
	Mutations status in *KRAS* codon 12/13	whole cohort (n=48)	11	19	3	15	0.79	0.44	0.61
	OT mini classifier cross validation	whole cohort (n=48)	14	2	0	32	**1.00**	**0.94**	**0.97**
EPO (n=60)	Mutation status in *KRAS/BRAF/NRAS*	whole cohort (n=60)	12	8	3	37	**0.80**	**0.82**	**0.81**
	Mutation status in *KRAS* codon 12/13	whole cohort (n=60)	12	20	3	25	**0.80**	0.56	0.68
	OT mini classifier	whole cohort (n=60)	14	5	2	39	**0.88**	**0.89**	**0.88**
	OT mini classifier	Wild-type for *KRAS* codon 12/13 (n=32)	11	3	1	17	**0.92**	**0.85**	**0.88**
	OT mini classifier	Wild-type for all *KRAS/BRAF/NRAS* (n=20)	11	3	1	5	**0.92**	0.63	0.77
NV (n=36)	Mutation status in *KRAS/BRAF/NRAS*	whole cohort (n=36)	5	11	3	17	0.63	0.61	0.62
	Mutation status in *KRAS* codon 12/13	whole cohort (n=36)	5	20	3	8	0.63	0.29	0.46
	OT mini classifier	whole cohort (n=36)	7	3	1	25	**0.88**	**0.89**	**0.88**
	OT mini classifier	Wild-type for *KRAS* codon 12/13 (n=25)	5	1	0	19	**1.00**	**0.95**	**0.98**
	OT mini classifier	Wild-type for all *KRAS/BRAF/NRAS* (n=16)	5	1	0	10	**1.00**	**0.91**	**0.95**
	Mutation status in *KRAS/BRAF/NRAS*	whole cohort excluding SD cases (n=32)	3	11	1	17	0.75	0.61	0.68
	Mutation status in *KRAS* codon 12/13	whole cohort excluding SD cases (n=32)	3	20	1	8	0.75	0.29	0.52
	OT mini classifier	whole cohort excluding SD cases (n=32)	4	3	0	25	**1.00**	**0.89**	**0.95**
	OT mini classifier	Wild-type for *KRAS* codon 12/13 & excluding SD cases (n=23)	3	1	0	19	**1.00**	**0.95**	**0.98**
	OT mini classifier	Wild-type for all *KRAS/BRAF/NRAS* & excluding SD cases (n=14)	3	1	0	10	**1.00**	**0.91**	**0.95**
KF (n=68)	Mutation status in *KRAS* codon 12/13	Cases with mutation information (n=59)	20	19	2	18	**0.91**	0.49	0.70
	OT mini classifier	whole cohort (n=68)	17	6	8	37	0.68	**0.86**	0.77
	OT mini classifier	Wild-type for *KRAS* codon 12/13 (n=39)	15	3	5	16	0.75	**0.84**	**0.80**
	Mutation status in *KRAS* codon 12/13	cases with mutation information & excluding SD cases (n=42)	5	19	0	18	**1.00**	0.49	0.74
	OT mini classifier	whole cohort excluding SD cases (n=49)	5	6	1	37	**0.83**	**0.86**	**0.85**
	OT mini classifier	Wild-type for *KRAS* codon 12/13 & excluding SD cases (n=24)	4	3	1	16	**0.80**	**0.84**	**0.82**

Supplementary Table 2: Performances of the OT mini-classifier and of the RAS/RAF mutation status in predicting cetuximab sensitivity in a merged superset of OT, EPO, NV and KF samples.

Merged cohorts	Classifier	Samples analyzed	TP	FP	FN	TN	Sensitivity	Specificity	Balanced accuracy
EPO+NV+KF (n=164)	Mutation status in *KRAS/BRAF/NRAS*	Cases with mutation information (n=96)	17	19	6	54	0.74	0.74	0.74
	Mutation status in *KRAS* codon 12/13	Cases with mutation information (n=155)	37	59	8	51	**0.82**	0.46	0.64
	OT mini classifier	whole cohort (n=164)	38	14	11	101	0.78	**0.88**	**0.83**
	OT mini classifier	Wild-type for *KRAS* codon 12/13 (n=96)	31	7	6	52	**0.84**	**0.88**	**0.86**
	OT mini classifier	Wild-type for all *KRAS/BRAF/NRAS* mutations (n=36)	16	4	1	15	**0.94**	0.79	**0.87**

Supplementary Table 3: List of primers used for validation of mutations.

Gene	AA change	Chr	Position	forward Primer	reverse Primer
SMAD4	A118V	18	48575159	TTTCTAGGTGGCTGGTCGGA	CAATTCCAGGTGATACAACTCGTTC
SMAD4	142_143del	18	48575665	TTGCCCCTTTAGAACATTGTTTTTG	TAATGTTACTGCCTGCCGCT
APC	R499X	5	112162891	TTAGGGGGACTACAGGCCAT	ACCTTGTTGGCTACATCTCCAAA
APC	914_914del	5	112174030	TCCAGGAACTTCTTCAAAGCGA	GTTTGAATGTGTATGGGCAGCAG
APC	A2690E	5	112179360	CAGAGGATGTTTGGGTGAGAATTG	TTTCCAAACCCACGGTACGC
APC	H1490fs	5	112175760	GGACCTAAGCAAGCTGCAGTAA	CATTTTCCTGAACTGGAGGCATT
PIK3CA	R88Q	3	178916876	AATACCCCCTCCATCAACTTCTTC	CTTTTCTTCACGGTTGCCTACTG
PIK3CA	E542K	3	178936082	TCTGTGAATCCAGAGGGGAAAAAT	CATTTTAGCACTTACCTGTGACTCC

Supplementary Table 4: Primers used for pentaplex PCR reactions to identify the microsatellite status.

Marker	Primer	Labeling
BAT25-forward	5'-TACCAGGTGGCAAAGGGCA-3'	HEX-labeled
BAT25-reverse	5'-TCTGCATTTTAACTATGGCTC-3'	-
BAT26-forward	5'-CTGCGGTAATCAAGTTTTTAG-3'	FAM-labeled
BAT26-reverse	5'-AACCATTCAACATTTTTAACCC-3'	-
NR21-forward	5'-GAGTCGCTGGCACAGTTCTA-3'	FAM-labeled
NR21-reverse	5'-CTGGTCACTCGCGTTTACAA-3'	-
NR24-forward	5'-GCTGAATTTTACCTCCTGAC-3'	TAMRA-labeled
NR24-reverse	5'-ATTGTGCCATTGCATTCCAA-3'	-
NR27-forward	5'-AACCATGCTTGCAAACCACT-3'	HEX-labeled
NR27-reverse	5'- CGATAATACTAGCAATGACC-3'	-

Supplementary Table 5: Doses and schedules of drug treatment of PDX.

Treatment	Application route	Schedule	Days	# of cycles	Weeks	Dose	Comments
Chemotherapy Oxaliplatin	i.p.	QDx5x2	Mo-Fr	2	1, 4	5 mg/kg	SoC: FOLFOX
Chemotherapy Irinotecan	i.p.	QDx5x2	Mo-Fr	2	1, 3	15 mg/kg	SoC: FOLFIRI
Chemotherapy 5-FU	i.p.	Q7Dx4	Mo	4	1- 4	100 mg/kg	SoC: FOLFOX, FOLFIRI
EGF Pathway Cetuximab	i.p.	BIW	Mo & Th	4	1- 4	30 mg/kg	EGFR MAB (Merck)
EGF Pathway AZD8931	p.o.	BID x28	Mo-Su	4	1- 4	25 mg/kg	Mixed EGFR/ERBB2/ERBB3 RTKI (AstraZeneca)
MEK Inhibitor Selumetinib	p.o.	BID x28	Mo-Su	4	1- 4	25 mg/kg	MEK (AstraZeneca)
EGF Pathway Afatinib	p.o.	QDx28	Mo-Su	4	1- 4	10 mg/kg (free base)	Dual irrev. EGFR RTKI (BI)
Angiogenese VEGF Avastin	i.p.	BIW	Mo & Th	4	1- 4	5 mg/kg	VEGF MAB (Roche)
Angiogenese VEGF Regorafenib	p.o.	QDx28	Mo-Su	4	1- 4	10 mg/kg	Multitarget RTKI (VEGFR, KIT) (Bayer)
Angiogenese VEGF Nintedanib	p.o.	QDx28	Mo-Su	4	1- 4	50 mg/kg	Multitarget RTKI (VEGFR, PDGF) (BI)
mTor Pathway mTOR FR	p.o.	QDx28	Mo-Su	4	1- 4	50 mg/kg (free base)	mTOR Inhibitor (BI)

Supplementary Table 6: Actin primers for bisulfite conversion covering 133bp (chr7: 5571729-5571861)

	Forward Primer	Reverse Primer
For bisulfite-converted DNA	5'-TGGTGATGGAGGAGGTTTAGTAAGT	5'-AACCAATAAAACCTACTCCTCCCTTAA
For unconverted DNA	5'-TGGTGATGGAGGAGGCTCAGCAAGT	5'-AGCCAATGGGACCTGCTCCTCCCTTGA
